# Notes and Memoranda for Hospital Buyers

**Published:** 1918-12-14

**Authors:** 


### DRUGS AFTER THE WAR.

### Notes and Memoranda for Hospital Buyers.

Sufficient time has elapsed since the signing of the armistice and the cessation of hostilities to enable us to form some idea how the values of drugs are likely to move in the near, as well as the more distant, future and how it would be advisable for institutional buyers to act in the circumstances. During the four and a quarter years in which the war continued we published week by week our usual note on the state of the drug market, giving where it was possible and when it seemed desirable a forecast of probable movements in price; and so far as we remember such forecasts as were given were unfailingly correct. This is said not as a boast, but merely as a statement of a fact which encourages us now to look further ahead.

When war broke out the public at large learnt for the first time how great was our dependence upon foreign and oversea sources for our supplies of the major portion of our *materia medica*. Hardly a pill, a potion, or a plaster could be compounded without paying toll to a foreigner or freight for the passage of one or all of the ingredients across the sea. Whether they were vegetable drugs like senna, or aloes, or sarsaparilla; whether they were synthetic products like antipyrine, phenacetin, or salol; or potash for making potash salts or bromine for making bromides, or waxes for making ointments or sugar for sweetening elixirs—most of them had to be brought to this country in ships.

The result was that when shipping began to be restricted, drugs began to get scarce. Where five ships used to arrive perhaps only one came, and that instead of making a voyage of three weeks took, say, four. Things of more material importance than drugs were wanted, so that the ship's space available for drugs was reduced to very narrow limits. Then, of course, freights rose to abnormal heights. We shall still be dependent on oversea sources for the bulk of our supplies of drugs, but as ships are released for ordinary mercantile traffic, and as they take a shorter time in passage, the empty spaces in our store-houses will in due course be filled up. Moreover, ships will be reasonably sure of arriving at their port of destination—a circumstance on which we certainly could not rely in 1917 and the best part of this year. Then freights will gradually be reduced—possibly not to pre-war levels for a long time to come; but that they will go down to competitive rates is obvious. Thus drugs will arrive in this country more frequently, more regularly, in larger quantities, and at a much lower transport cost.

What about the conditions in the countries of production? It is commonly known that at the source of supply many products are simply "eating their heads off" because there are no ships to take them away. It is true that in many places labour will be scarce for a long time ahead and will—let us hope it will—always be dearer than it was before the war, but even if the collectors, packers, and porters are paid a reasonable wage the addition to the cost would be trivial compared with the additions made by the grower and the many middlemen who intervene between him and the consumer. All this means to say that vegetable drugs—practically all vegetable drugs—are bound to dwindle in price from now onwards until the natural law of supply and demand comes into its own again.

The profiteer is a product of necessary war-time restrictions on output; with the dams removed from sources of supply and markets unrestricted the old game of "beggar my neighbour" will begin once again—the players paradoxically observing the rule of the game that each must make a living—and it will be possible by a simple process in mathematics to calculate what the price in London ought to be of a drug grown, say, in California. During the war the size of the crop bore not the remotest relationship to the market value, an obviously unnatural thing. As crude drugs become less costly and the materials used in extracting their active principles become freely available, we may expect to see lower prices for alkaloids and galenical preparations.

Turning to drugs other than those of vegetable origin, take synthetic products. Things like aspirin, antipyrin, salicylate of soda, phenacetin, and salol, the consumption of which is extraordinarily large, were made almost exclusively in Germany before the war. Now these are being made all over the world—Japan is making them, England is making them. The prices are still very high, and there is room for a big drop. That this drop will come is just as sure as that the Germans have lost the war; the raw materials used for the manufacture of these products are no longer wanted for making explosives. America, Great Britain, Switzerland, France, Japan, and other countries will all be offering on the world's markets synthetic drugs made from unrestricted supplies of cheap raw material. It would require a miracle to keep the prices from falling. Take another class of drugs. The supply of potash will be more abundant than ever it was; potash salts will dwindle. Quicksilver will in due course be released from Government control, and makers of calomel and other salts of mercury will be assured of supplies restricted only by output of metal. And so we might go through the whole gamut. Drugs may never be as cheap as they were before the war—they may be cheaper—these are questions time will answer. But we can be sure that they will soon be much cheaper than they are now.

